# Supplementary material for: Diversity in medullary thymic epithelial cells controls the activity and availability of iNKT cells
Source: Nat Commun. 2020 May 4;11:2198. doi: 10.1038/s41467-020-16041-x (PMC7198500; doi:10.1038/s41467-020-16041-x)
Supplement: Supplementary file 1 — Supplementary Information [file 41467_2020_16041_MOESM1_ESM.pdf]

Supplementary Information

Diversity in Medullary Thymic Epithelial Cells Controls The Activity and Availability of iNKT cells. Lucas et al

Lucas et al Supplementary Figure 1

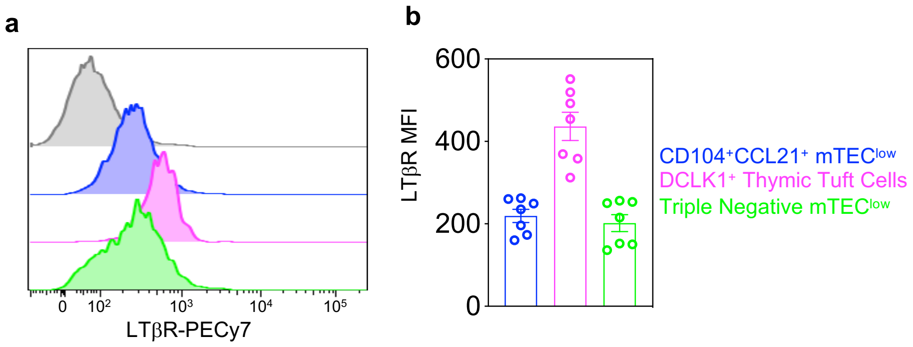

Supplementary Figure 1. Expression of LTβR By $mTEC^{low}$ Subsets.

(a) Digested adult WT thymus, with total $mTEC^{low}$ ($EpCAM1^{+}Ly51^{-}UEA1^{+}CD80^{low}MHCII^{low}$) cells analysed by flow cytometry for cell surface expression levels of LTβR. Magenta indicates $DCLK1^{+}$ thymic tuft cells, blue indicates $CD104^{+}CCL21^{+}$ $mTEC^{low}$, and green indicates $CCL21^{-}CD104^{-}DCLK1^{-}$ $mTEC^{low}$. Grey histograms indicate LTβR expression in total mTEC from $LT\beta R^{TEC}$ mice. (b) Graph shows mean MFI of LTβR expression in $CD104^{+}CCL21^{+}$, $DCLK1^{+}$, and $CD104^{-}CCL21^{-}DCLK1^{-}$ $mTEC^{low}$ subsets, error bars represent SEM, n=7 independent biological samples, over 3 independent experiments. Source data are provided as a Source Data file.

Lucas et al Supplementary Figure 2

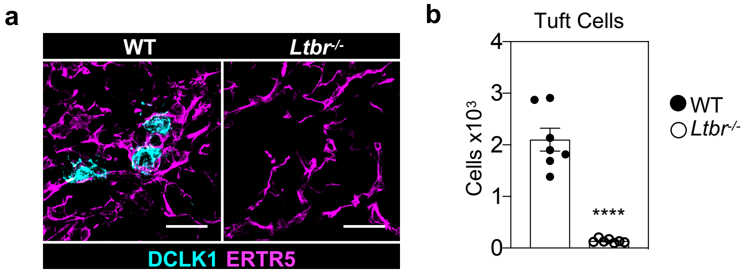

Supplementary Figure 2. Thymic Tuft Cells Are Absent In Germline *Ltbr*$^{-/-}$ Mice

(a) Frozen thymus sections from WT and germline *Ltbr*$^{-/-}$ adult mice were labelled with ERTR5 antibody to detect mTEC (magenta), and anti-DCLK1 to detect thymus tuft cells (cyan). Scale bar denotes 20μm. Images are representative of at least 3 mice of each strain. (b) Intracellular flow cytometric staining of mTEC^{low} from WT and *Ltbr*$^{-/-}$ mice for expression of DCLK1 was performed, and DCLK1^{+} cells enumerated. Graphs indicate absolute cell numbers in WT mice and *Ltbr*$^{-/-}$ mice, n=7 independent biological samples, over 3 independent experiments. Significant P values using two-tailed unpaired t-test as follows: No. Tuft cells p=<0.0001. All data is represented as mean +/- SEM. **** P<0.0001. Source data are provided as a Source Data file.

Lucas et al Supplementary Figure 3

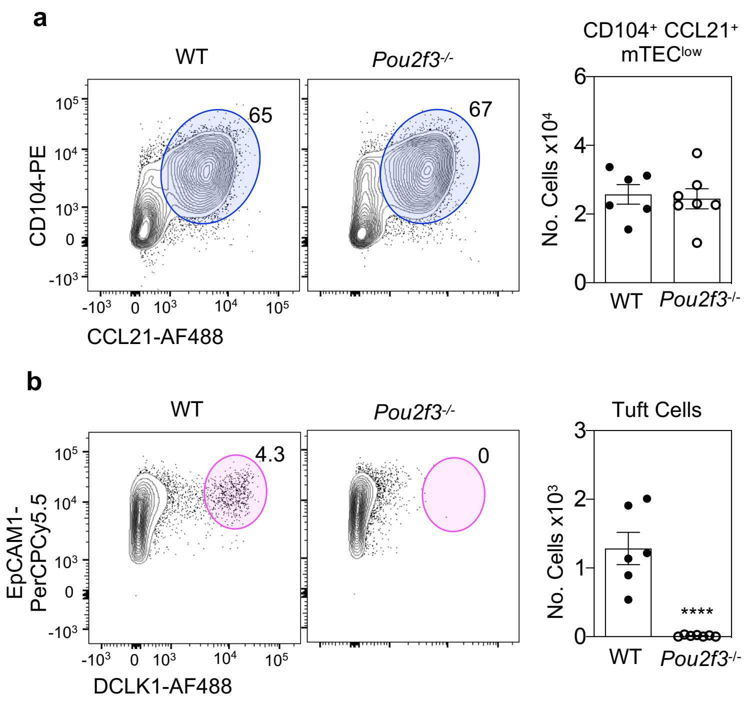

Supplementary Figure 3. DCLK1$^+$ Thymic Tuft Cells, But Not CD104$^+$CCL21$^+$ mTEC^{low}, Are Absent In *Pou2f3$^{-/-}$* Mice.

(a) Thymus tissue from adult WT and *Pou2f3$^{-/-}$* mice was digested and mTEC^{low} heterogeneity analysed by flow cytometry. FACS plots indicate CD104$^+$CCL21$^+$ mTEC^{low} (blue) and DCLK1$^+$ thymic tuft cells (magenta). Bar graphs in (b) show absolute numbers of thymic tuft cells and CD104$^+$CCL21$^+$ mTEC^{low} in WT (n=6 independent biological samples), and *Pou2f3$^{-/-}$* (n=7 independent biological samples) mice, over 3 independent experiments. Significant P values using two-tailed unpaired t-test as follows: No. Tuft cells p=0.0001. All data is represented as mean +/- SEM. **** P<0.0001. Source data are provided as a Source Data file.

Lucas et al Supplementary Figure 4

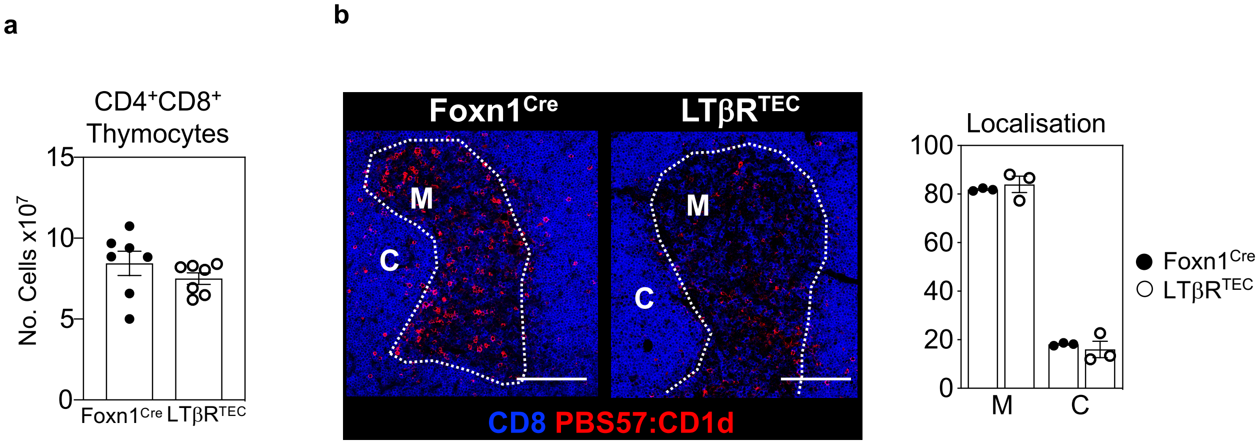

Supplementary Figure 4. iNKT Cells Locate To The Thymus Medulla in LTβR^{TEC} Mice.

(a) Numbers of CD4^{+}CD8^{+} thymocytes in adult Foxn1^{Cre} controls and LTβR^{TEC} mice were determined by flow cytometry, n=7 independent biological samples, over 3 independent experiments. (b) Whole thymuses from adult Foxn1^{Cre} controls (n=3 independent biological samples) and LTβR^{TEC} (n=3 independent biological samples) were incubated in PBS57:CD1d tetramer as described in materials and methods. Cryosections were stained to amplify PBS57:CD1d tetramer staining (red), and co-stained with anti-CD8 (blue) to reveal cortical (C) and medullary (M) areas. Scale bar denotes 50μm. The bar graph shows quantitation of the location of CD1d-tetramer^{+} cells in thymus sections of both strains, represented as mean +/- SEM. Cells were enumerated in 100mm x 100mm areas within the cortex and medulla. At least three medullary and three cortical regions were analysed per section. Four sections were analysed per mouse, obtained from differing depths throughout the thymus. All data is represented as mean +/- SEM. Source data are provided as a Source Data file.

Lucas et al Supplementary Figure 5

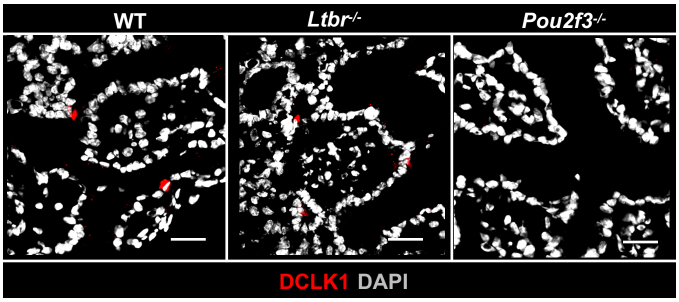

Supplementary Figure 5. Intestinal Tuft Cells Are Present *Ltbr*$^{-/-}$ mice.

Cryosections of small intestine from WT, LTβR$^{-/-}$ and Pou2f3$^{-/-}$ mice were stained for DCLK1 (red), and counterstained with DAPI (grey). Scale bar denotes 20µm. Images are representative of 3 mice of each genotype.

Lucas et al Supplementary Figure 6

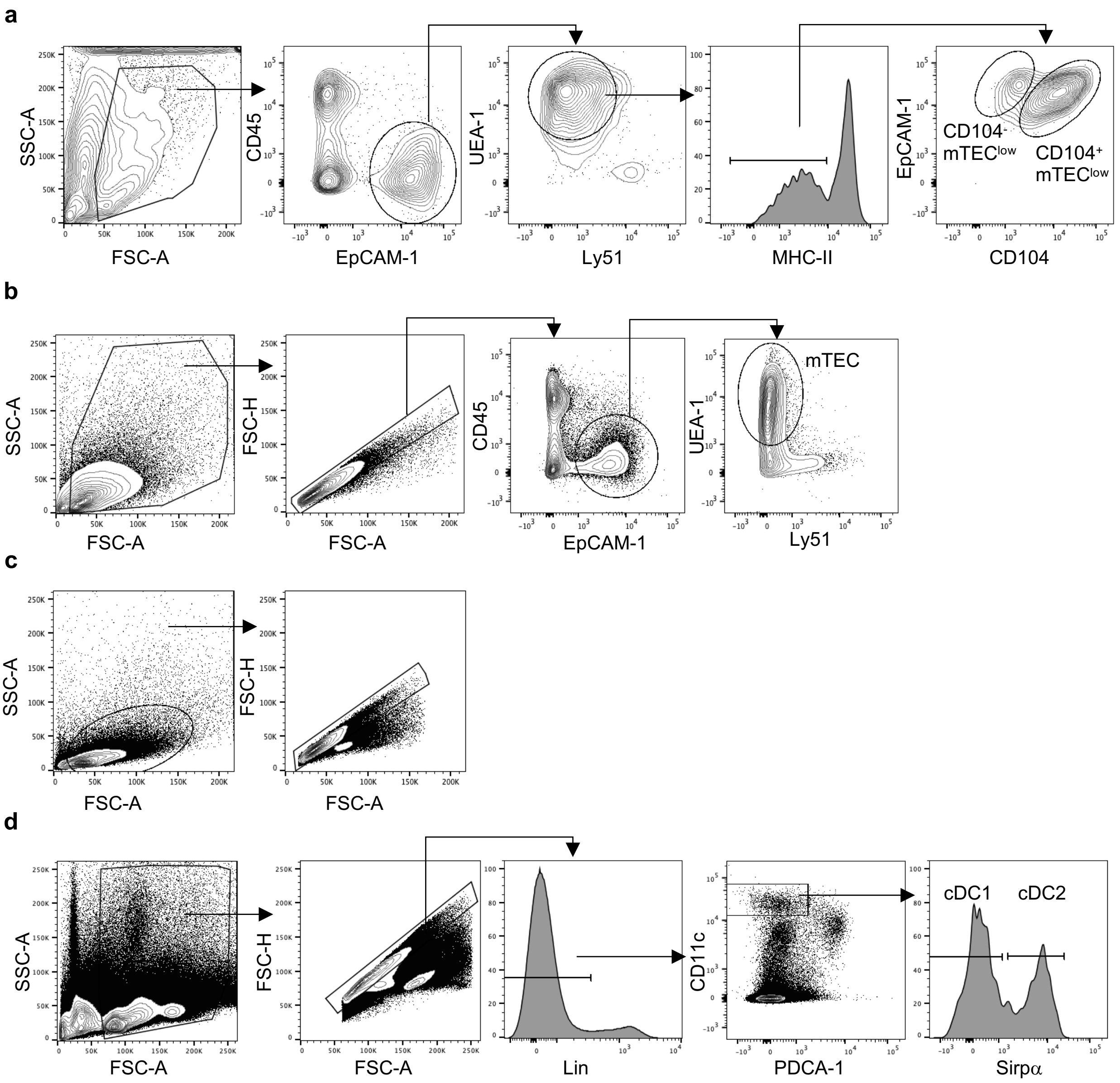

Supplementary Figure 6. Gating Strategies Used For Cell Sorting and Flow Cytometry

(a) Sorting strategy used to obtain purified populations of CD104$^+$ and CD104$^-$ mTEC^{low} in Fig 1b, 2c, 3e, 3f. (b) Gating strategy used to identify mTEC in Fig 1a, 1d, 2a, 2d, 3a, 3b. (c) Gating strategy used to gate on live lymphocytes prior to iNKT cell gating in Fig 3a, 3c, 3d, 4a, 4c, 4d, 6b, 8a. (d) Gating strategy used to identify cDC1 and cDC2 in Fig 7a, 7b, ‘Lin’ includes CD3, CD19, NK1.1.
