# Supplementary material for: Diversity in medullary thymic epithelial cells controls the activity and availability of iNKT cells
Source: Nat Commun. 2020 May 4;11:2198. doi: 10.1038/s41467-020-16041-x (PMC7198500; doi:10.1038/s41467-020-16041-x)
Supplement: Supplementary file 3 — Reporting Summary [file 41467_2020_16041_MOESM3_ESM.pdf]

nature research

Corresponding author(s): Graham Anderson

Last updated by author(s): Apr 8, 2020

Reporting Summary

Nature Research wishes to improve the reproducibility of the work that we publish. This form provides structure for consistency and transparency in reporting. For further information on Nature Research policies, see Authors & Referees and the Editorial Policy Checklist.

Statistics

For all statistical analyses, confirm that the following items are present in the figure legend, table legend, main text, or Methods section.

n/a	Confirmed	
☐	☒	The exact sample size (n) for each experimental group/condition, given as a discrete number and unit of measurement
☐	☒	A statement on whether measurements were taken from distinct samples or whether the same sample was measured repeatedly
☐	☒	The statistical test(s) used AND whether they are one- or two-sided<br>*Only common tests should be described solely by name; describe more complex techniques in the Methods section.*
☐	☒	A description of all covariates tested
☒	☐	A description of any assumptions or corrections, such as tests of normality and adjustment for multiple comparisons
☐	☒	A full description of the statistical parameters including central tendency (e.g. means) or other basic estimates (e.g. regression coefficient) AND variation (e.g. standard deviation) or associated estimates of uncertainty (e.g. confidence intervals)
☐	☒	For null hypothesis testing, the test statistic (e.g. F, t, r) with confidence intervals, effect sizes, degrees of freedom and P value noted<br>*Give P values as exact values whenever suitable.*
☒	☐	For Bayesian analysis, information on the choice of priors and Markov chain Monte Carlo settings
☒	☐	For hierarchical and complex designs, identification of the appropriate level for tests and full reporting of outcomes
☒	☐	Estimates of effect sizes (e.g. Cohen's d, Pearson's r), indicating how they were calculated

Our web collection on statistics for biologists contains articles on many of the points above.

Software and code

Policy information about availability of computer code

Data collection: Not applicable

Data analysis: Not applicable

For manuscripts utilizing custom algorithms or software that are central to the research but not yet described in published literature, software must be made available to editors/reviewers. We strongly encourage code deposition in a community repository (e.g. GitHub). See the Nature Research guidelines for submitting code & software for further information.

Data

Policy information about availability of data

All manuscripts must include a data availability statement. This statement should provide the following information, where applicable:

- Accession codes, unique identifiers, or web links for publicly available datasets
- A list of figures that have associated raw data
- A description of any restrictions on data availability

All data relevant to this manuscript is contained within the submitted figures

Field-specific reporting

Please select the one below that is the best fit for your research. If you are not sure, read the appropriate sections before making your selection.

☒ Life sciences ☐ Behavioural & social sciences ☐ Ecological, evolutionary & environmental sciences

For a reference copy of the document with all sections, see nature.com/documents/nr-reporting-summary-flat.pdf

October 2018

Life sciences study design

All studies must disclose on these points even when the disclosure is negative.

Sample size	Sample size for each of our experiments is shown in the relevant figure legends. This was chosen based on previous experience with similar experiments, and in order to reproducibly detect specific effects. Statistical methods were not used to predetermine sample size.
Data exclusions	No data was excluded from analysis.
Replication	All experiments were repeated so data in the manuscript are from at least three or four independent experiments that gave similar results. The figure legends contain exact details of the numbers of repeats for each experiment.
Randomization	No randomization of mice was performed. Mice that were used in all experiments were litter mates, age and sex-matched where possible.
Blinding	Investigators were not blinded to mouse genotypes in our experiments. This was to help ensure that appropriate sample size was achieved by sacrificing the minimum number of mice required to achieve consistent data.

Reporting for specific materials, systems and methods

We require information from authors about some types of materials, experimental systems and methods used in many studies. Here, indicate whether each material, system or method listed is relevant to your study. If you are not sure if a list item applies to your research, read the appropriate section before selecting a response.

Materials & experimental systems

n/a	Involved in the study	
☐	☒	Antibodies
☒	☐	Eukaryotic cell lines
☒	☐	Palaeontology
☐	☒	Animals and other organisms
☒	☐	Human research participants
☒	☐	Clinical data

Methods

n/a	Involved in the study	
☒	☐	ChIP-seq
☐	☒	Flow cytometry
☒	☐	MRI-based neuroimaging

Antibodies

Antibodies used	anti-TCR (H57-597, eBioscience, Cat No.: 47-5961-82, 1:200), anti-PLZF (Mags.21F7, eBioscience, Cat No.: 53-9320-82, 1:100), anti-RORt (Q31-378, Becton Dickinson, Cat No.: 564723, 1:100), anti-T-bet (4B10, eBioscience, Cat No.: 50-5825-82, 1:100), anti-T-bet (4B10, Biolegend, Cat No.: 644820, 1:100), anti-CD122 (TM-b1, eBioscience, Cat No.: 25-1222-82, 1:100), anti-IL17RB (MUNC33, eBioscience, Cat No.: 12-7361-82, 1:100), anti-CD45 (30-F11, eBioscience, Cat No.: 47-0451-82, 1:1000), anti-EpCAM1 (G8.8, eBioscience, Cat No.: 46-5791-82, 1:2000), anti-Ly51 (6C3, eBioscience, Cat No.: 17-5891-82, 1:1000), anti-IA/IE (M5/114.15.2, eBioscience, Cat No.: 56-5321-82, 1:2000), anti-CD80 (16-10A1, BioLegend, Cat No.: 104729, 1:800), anti-CD104 (346-11A, BioLegend, Cat No.: 123610, 1:1000). Biotinylated UEA-1 (Vector laboratories, B-1065, 1:10,000), Streptavidin PECy-7 (eBioscience, Cat No.: 25-4317-82, 1:1500), anti-CCL21 (Lifespan Biosciences, Cat No.: LS-C104634, 1:100) and anti-DCLK1 (DCAMKL1, Abcam, Cat No.: Ab31704, 1:1000), donkey anti-rabbit Alexa Fluor 488 (Invitrogen, Cat No.: A-21206, 1:1000), anti-CD11c (N418, eBioscience, Cat No.: 25-0114-82, 1:800), anti-PDCA-1 (129C1, BioLegend, Cat No.: 127018, 1:200), anti-Sirp (P84, eBioscience, Cat No.: 12-1721-82, 1:200), anti-IA/IE (M5/114.15.2, eBioscience, Cat No.: 56-5321-82, 1:200), anti-CD86 (GL1, BioLegend, Cat No.: 105035, 1:200). Anti-CD3 (145-2C11, eBioscience, Cat No.: 47-0031-82, 1:200), anti-CD19 (eBio1D3, eBioscience, Cat No.: 47-0193-82, 1:200), anti-NK1.1 (PK136, eBioscience, Cat No.: 47-5941-82, 1:200), anti-Bcl2 (BCL/10C4, BioLegend, Cat No.: 635510, 1:100), anti-Ki67 (SolA15, eBioscience, Cat No.: 25-5698-82, 1:2000), anti-CD8 (53-6.7, BioLegend, Cat No.: MCD0821, 1:100), ERTR5 (W. van. Ewijk), donkey anti-rabbit Alexa Fluor 555 (Invitrogen, Cat No.: A-31572, 1:1000) and goat anti-rat IgM Alexa Fluor 488 (Invitrogen, Cat No.: A-11006, 1:200), rabbit anti-PE (Invitrogen, Cat No.: 12-4739-81, 1:100), donkey anti-rabbit Alexa Fluor 555 (Invitrogen, Cat No.: A-31572, 1:1000), anti-IL4 (11B11, eBioscience, Cat No.: 17-7041-82, 1:100), anti-IFNg (XMG1.2, BioLegend, Cat No.: 505813, 1:100)
Validation	All antibodies were optimised for flow cytometry and confocal microscopy by titration prior to use in all experiments.

Animals and other organisms

Policy information about studies involving animals; ARRIVE guidelines recommended for reporting animal research

Laboratory animals	Species: Mus Musculus. All mice were used at age 8-12 weeks unless stated otherwise. Strains used:<br>Wildtype (WT) C57BL/6 , B6 Pou2f3-/-, germline Ltbr-/-, B6 Cd1d1-/- CCL21tdTOM, LTRTEC mice (generated by crossing Foxn1Cre mice with LTbRfl/fl mice.

Wild animals	Not applicable
Field-collected samples	Not applicable.
Ethics oversight	All mouse experiments were done with approval from the University of Birmingham Animal Welfare and Ethical Review Body and the UK Home Office.

Note that full information on the approval of the study protocol must also be provided in the manuscript.

Flow Cytometry

Plots

Confirm that:

☒ The axis labels state the marker and fluorochrome used (e.g. CD4-FITC).

☒ The axis scales are clearly visible. Include numbers along axes only for bottom left plot of group (a 'group' is an analysis of identical markers).

☒ All plots are contour plots with outliers or pseudocolor plots.

☒ A numerical value for number of cells or percentage (with statistics) is provided.

Methodology

Sample preparation	All samples were prepared from freshly isolated mouse organs by either mechanical disruption or enzymatic digestion.
Instrument	Becton Dickinson Fortessa Flow Cytometer
Software	FACS DIVA software (BD Biosciences) and FlowJo software (TreeStar)
Cell population abundance	All FACS sorted populations were sorted to a purity of 95% or greater
Gating strategy	Viable cells were identified by flow cytometry using FSC/SSC gating. Discrimination of cells staining postively or negatively for antibodies was achieved by gating on samples stained with either isotype controls, or following omission of the primary antibody.

☒ Tick this box to confirm that a figure exemplifying the gating strategy is provided in the Supplementary Information.
